# A systematic review of automated journalism scholarship: guidelines and suggestions for future research

**DOI:** 10.12688/openreseurope.13096.1

**Published:** 2021-03-24

**Authors:** Samuel Danzon-Chambaud

**Affiliations:** 1 School of Communications, Dublin City University, Dublin, Ireland

**Keywords:** automated journalism, robot journalism, algorithmic journalism, computational journalism, natural language generation, automation, journalism

## Abstract

**Background: **The use of advanced algorithmic techniques is increasingly changing the nature of work for highly trained professionals. In the media industry, one of the technical advancements that often comes under the spotlight is automated journalism, a solution generally understood as the auto generation of journalistic stories through software and algorithms, without any human input except for the initial programming.

**Methods: **In order to conduct a systematic review of existing empirical research on automated journalism, I analysed a range of variables that can account for the semantical, chronological and geographical features of a selection of academic articles as well as their research methods, theoretical backgrounds and fields of inquiry. I then engaged with and critically assessed the meta-data that I obtained to provide researchers with a good understanding of the main debates dominating the field.

**Results: **My findings suggest that the expression “automated journalism” should be called into question, that more attention should be devoted to non-English speaking scholarship, that the collective and individual impacts of the technology on media practitioners should be better documented and that well-established sociological theories such as institutionalism and Bourdieu’s field theory could constitute two adequate frameworks to study automated journalism practices.

**Conclusions:** This systematic literature therefore provides researchers with an overview of the main challenges and debates that are occurring within the field of automated journalism studies. Future studies should, in particular, make use of institutionalism and field theory to explore how automated journalism is impacting the work of media practitioners, which could help unearth common patterns across media organisations.

## Introduction

The use of advanced algorithmic techniques is increasingly changing the nature of work for highly trained professionals. In the medical industry, health professionals can rely on neural networks to detect skin cancers among patients ( Esteva *et al*., 2017) while advanced classification or natural language processing methods can be deployed to predict judgements in the legal industry ( Katz *et al.*, 2017; Medvedeva *et al.*, 2020). In the media industry, one of the technical advancements that often comes under the spotlight is automated journalism, a solution generally understood as the auto generation of journalistic stories through software and algorithms, with no human intervention except for the initial programming ( Graefe, 2016). Also popularised under the terms “robot journalism” ( Lemelshtrich Latar, 2018), “algorithmic journalism” ( Dörr, 2016) and “machine-written news” ( van Dalen, 2012), automated journalism builds on natural language generation (NLG), a computer process that triggers text generation. Employed for several decades in domains such as sports, finances and weather forecasting ( Dörr, 2016), NLG systems traditionally involve pre-written templates that are filled through a set of specific rules, while other methods rely on machine learning techniques to “learn patterns of language use from large corpora of examples” ( Diakopoulos, 2019, p. 101; Graefe, 2016).

Automated journalism started to be more widely discussed in the 2010s as The Los Angeles Times started covering homicides in an automated fashion and launched a tool to generate earthquake alerts, while The Associated Press partnered with the firm Automated Insights to automate the majority of its corporate earnings stories. At the same time, The Washington Post developed an in-house software to produce short automated stories and alerts during the 2016 Rio Olympics. In France, Le Monde collaborated with the firm Syllabs to automatically cover the results of the 2015 regional elections while, in Switzerland, Tamedia Group used Automated Insights’ solution to report on the outcome of a 2018 referendum ( Plattner & Orel, 2019). The BBC also resorted to a form of semi-automated journalism to cover the results of the 2019 United Kingdom general election. In addition to this, a dozen of European news agencies adopted or planned for the development of the technology ( Fanta, 2017).

At the same time, automated journalism is also increasingly attracting the attention of the academic community. The algorithmic nature of automated journalism turns it into a prime candidate for the study of algorithmic decision-making, an area of research that looks into tasks and processes commanded by algorithms. As such, the technology is sometimes discussed along with algorithmic distribution of media content ( Carlson, 2018; Napoli, 2014). Automated journalism can also be investigated as being part of computational journalism studies ( Bucher, 2017; Coddington, 2015), a discipline that initially focused on the use of advanced software to assist journalists with their daily workflow ( Cohen *et al*., 2011; Flew *et al*., 2012; Hamilton & Turner, 2009) and then expanded to journalists’ abilities to solve problems through abstraction and computing skills ( Gynnild, 2014; Stavelin, 2013). It is for this reason that automated journalism is sometimes only mentioned as “computational journalism” ( Lindén, 2017; Young & Hermida, 2015). Finally, automated journalism can be studied in the context of newsroom automation and artificial intelligence in journalism, a discipline that looks into the latest applications of computational breakthroughs in the media industry, such as platforms handling the distribution of news on social media and data-mining techniques for investigative journalism ( Diakopoulos, 2019; Marconi, 2020).

This systematic literature review analyses the key features of a selection of academic articles on automated journalism in order to provide a comprehensive overview of the field, and to contribute guidelines and suggestions for future research endeavours. Although Graefe & Bohlken (2020) conducted a meta-analysis focusing on readers’ perceptions of automated journalism, no scholarship has so far assessed the full range of peer-reviewed journal articles contributing empirical evidence to this growing research area. My research questions are therefore as follows:

1.   Which patterns arise from a selection of key variables used in a corpus of peer-reviewed articles on automated journalism that are systematically retrieved?
2.   Based on this pattern analysis, what are the main debates dominating the field of automated journalism studies?

## Methods

### Selection of corpus

To carry out this review, I retrieved the documents that constituted the final corpus in a systematic manner, with the following combined search query: "automated journalism" OR "algorithmic journalism" OR "robot journalism" OR "machine-written journalism" OR "computational journalism." I searched through five databases (Taylor and Francis, Sage, ScienceDirect, SpringerLink, and Scopus) as I looked for content published between 2005 and 2020 to represent the last 15 years of research, a range that shall accommodate the purpose of this research as it goes back five years prior to the launch of The Los Angeles Times’ pioneering project on automating homicide coverage.

Out of close to 500 results, I only selected articles written in English, that are based on empirical research to assess the latest findings in the field, and that presented an exclusive focus on automated journalism, that I understood as “the process of using software or algorithms to automatically generate news stories without human intervention – after the initial programming of the algorithm” ( Graefe, 2016, p. 14), a definition that I also extended to auto generated text so as to account for recent developments in the field ( Lindén, 2017). In the end, this final corpus was constituted of 33 scholarly articles on automated journalism ( Danzon-Chambaud, 2021a), a number that I am satisfied with, given that automated journalism was introduced into newsrooms and attracted scholars’ attention only recently. This number involves almost three times as many articles as in Graefe’s and Bohlken’s meta-analysis of readers’ perceptions of the technology (2020) and comes close to the 40 articles analysed in an authoritative systematic review of data journalism scholarship ( Ausserhofer *et al*., 2017), which constitutes another major development in the field of media and communication studies.

 Then, in order to conduct an efficient review of the field, I looked, first, for variables I could quantitatively measure, such as the semantical, chronological and geographical features of the articles studied as well as their research methods, fields of inquiry and theoretical backgrounds. To do this, I retrieved every keyword mentioned in the corpus, the years the articles were published online as well as the countries they originated from, the domains of investigation and methods used in each article, and lastly, the theoretical considerations and any bibliographic reference cited more than five times in the entire corpus. Second, I engaged with and critically assessed these meta-data in order to equip researchers with a more qualitative understanding of the main debates dominating the field. No risk of bias was found, apart from limiting my search to articles only written in English, a point that I address in the study. 

## Results

### “Robot journalism” vs. “automated journalism”

**Figure 1.**
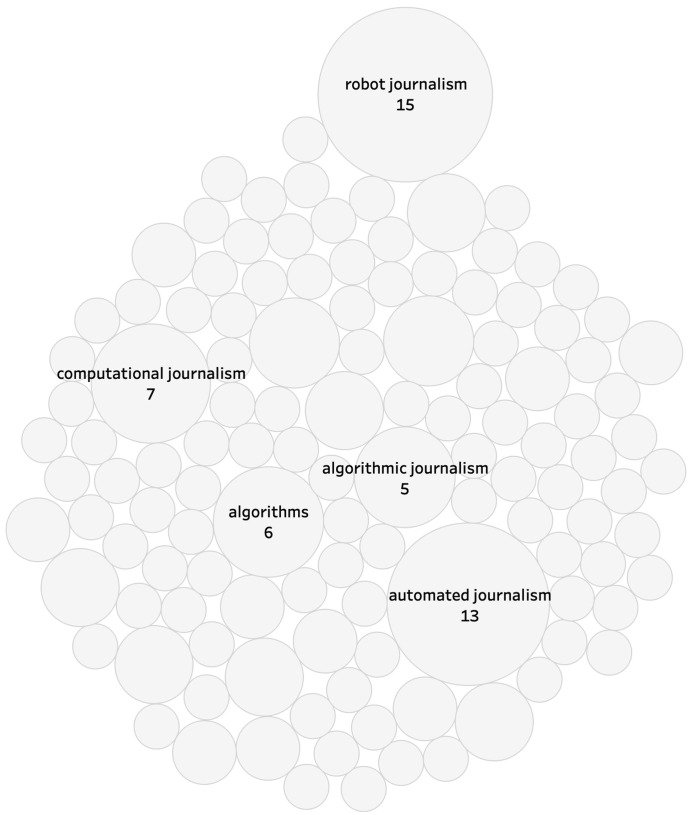
Most-frequent keywords used in corpus. Only keywords mentioned 5 times or more are indicated.

In order to investigate the various semantics used in the field of automated journalism, I first analysed the different keywords mentioned in the corpus ( Figure 1). I found that the most-frequently used ones referred to the terms “robot journalism” and “automated journalism”, two expressions that are regularly employed in mainstream media and academia to evoke the computer-generation of news text, but which both face criticisms related to their exact meaning. 

 The most problematic use of the term “robot journalism” lies in the fact that NLG involves a computer script and no actual robot ( Dörr, 2016). Lindén (2017, p. 125) cautions against a “popular but banal conceptualisation where illustrators more often portray robots writing on computer keyboards,” that ultimately plays on journalists’ fears of being made redundant and prevents newsrooms from being more innovative ( Lindén & Dierickx, 2019). This metaphor could also be detrimental to the acceptance of automated stories, since readers can ultimately feel deceived after being drawn to believe that the technology exhibits a form of humanness ( Waddell, 2018). In addition, there could be no added benefit to using this expression since readers have been revealed to be equally receptive to automated news labelled as a product of algorithms or identified through software name ( Waddell, 2019a). Only readers that were previously exposed to robots in popular culture seem to perceive this metaphor in a more positive light ( Waddell, 2018).

That being said, the use of the alternate expression “automated journalism” could also be up for debate. Although it is increasingly a much-preferred term within industry and academia, it is argued that its focus on computer-generated text is too narrow and does not reflect media practitioners' views. In fact, Wu *et al.* (2019b, p. 1453) advance that the definition of “automated journalism” could be extended to a whole other range of tasks, which could include, for instance, “anything from the machine aggregating and funnelling of content, to data scraping and auto-publication of stories”.

If the use of the “robot journalism” metaphor has largely been called into question, the use of the expression “automated journalism” – that I employ here because it is less controversial among scholars – has rarely been interrogated in the same manner. It should nevertheless be reflected upon, since this term could also encompass other algorithmic tasks in journalism such as retrieving newsworthy data in investigative reporting ( Broussard, 2015; Stray, 2019) or using algorithms to automate fact-checking ( Graves, 2018). Besides, the use of other popular keywords such as “computational journalism” and “algorithmic journalism” could not really supplant “automated journalism” as they do have an equally broad meaning, leaving room for discussion among scholars. 

### Looking beyond the Anglosphere

The second type of variables I looked for were the years the articles were published online and the countries from where research originated ( Table 1). While no publication was found prior to 2012, a steady growth in the number of articles published on automated journalism started to be noticeable from 2014 onwards, with the only exception of a small decrease in 2015 and an incomplete picture in 2020 since I finalised my data collection in mid-June of that year (it is also likely that research published in 2020 will be impacted by the coronavirus outbreak). This growth can be interpreted as a reflection of the adoption of the technology, since major media outlets announced their passage to automated journalism at about the same time. Looking at the countries from which the research originated, the results were not surprising as I limited my search to English-written scholarship; English-speaking countries (i.e. United States, Australia, Canada, United Kingdom) constituted the largest group in my ranking, followed by other western countries (i.e., Germany, Denmark, Finland, Israel, Netherlands, Spain, Sweden, Switzerland) and Asian countries (South Korea, India, Singapore). No scholarship associated with an African country or a South American country were found, but this probably was to do with my focus on scholarship written in English than with technology penetration in developing countries, though this is not negligible. As a matter of fact, the 2019 conference of the International Association for Media and Communication Research featured a few papers on automated journalism from African ( Ojomo & Ikem, 2019; Salawu, 2019) and South American ( Limia, 2019) countries, which should result, sooner rather than later, in more publications from these areas. 

**Table 1.** Articles' online publication years and countries of origin.

Country	2012	2013	2014	2015	2016	2017	2018	2019	2020	Grand Total
Australia	–	–	–	–	–	–	–	1	–	**1**
Canada	–	–	1	–	–	–	–	–	–	**1**
Denmark	1	–	–	–	–	–	–	–	–	**1**
Germany	–	–	–	–	1	1	–	–	–	**2**
Finland	–	–	–	–	1	–	1	–	–	**2**
Great Britain	–	–	–	–	–	–	–	1	–	**1**
India	–	–	–	–	–	–	1	–	–	**1**
Israel	–	–	–	–	1	–	–	–	–	**1**
Netherlands	–	–	–	–	–	–	1	–	–	**1**
Singapore	–	–	–	–	–	–	–	–	1	**1**
South Korea	–	–	–	–	1	2	1	–	–	**4**
Spain	–	–	–	–	–	–	–	2	1	**3**
Sweden	–	–	1	–	–	–	–	–	–	**1**
Switzerland	–	–	–	1	–	–	–	–	–	**1**
United States	–	–	1	1	–	2	2	3	–	**9**
China-United States	–	–	–	–	–	–	1	–	–	**1**
Germany-Switzerland	–	–	–	–	–	1	–	–	–	**1**
Switzerland-United States	–	–	–	–	–	1	–	–	–	**1**
**Grand Total**	**1**	**0**	**3**	**2**	**4**	**7**	**7**	**7**	**2**	**33**

Having made this observation, it is once I combined the online publication dates with the countries of origin that I started to notice some more patterns. First and foremost, two out of four articles published between 2012 and 2014 originated from Northern European countries (i.e., Denmark and Sweden) while the other half came from Canada and the United States. These two articles from Northern Europe could be considered to be pioneering work on automated journalism since they first tackled its impacts on media practitioners ( van Dalen, 2012) and the perceptions triggered among audiences ( Clerwall, 2014). These findings also suggest that Northern European outlets had a key role to play in the development of automated journalism, although much of the spotlight was on – and remains with – large media organisations in the United States, such as The Los Angeles Times, The Associated Press and The Washington Post. We can look for instance at Danish financial news agency Ritzau and Swedish media group MittMedia, which have both been engaging with automated journalism as early as 2015. Since they either partially or entirely owned the solution they launched ( Falk Eriksen, 2018; Lindén & Tuulonen, 2019), Ritzau and MittMedia differ from other media organisations such as Le Monde and The Associated Press, which adopted automated journalism at about the same time but outsourced its development to external NLG providers.

The combination of online publication years and countries of origin also showed a surge of research coming from East, South and Southeast Asia from 2016 onwards. While most of them were concerned with South Korea ( Jung *et al*., 2017; Kim & Kim, 2017; Kim & Kim, 2018; Kim & Lee, 2018), others originated from India ( Visvam Devadoss, 2019), Singapore ( Tandoc *et al.*, 2020) and partly from China ( Zheng *et al.*, 2018). We can assume that this picture only represents the “tip of the iceberg,” as it is likely that additional research has been published in local languages I could not read. In the case of China, for instance, only a handful of information is readily available in English; it is known that news agencies Xinhua and Toutiao as well as media group Caixin are resorting to automated journalism, that the technology is used for the same kind of reporting than in Western outlets (i.e., sports, financial news, weather), and that at least one Chinese firm, Tencent, acts as an NLG provider ( Dörr, 2016; Lindén & Tuulonen, 2019). This scarcity of materials written in English prevents me from knowing more about the strategies these organisations develop. Little is known about the “Media Brain” project that China’s state agency Xinhua launched in 2018. Initially described as “a first-of-its-kind platform in China that brings cloud computing, the Internet of Things, Big Data and AI technology into news production,” it has also reinforced suspicions about the way AI could be used to further disseminate propaganda coming from the Chinese Communist Party. 

In sum, pioneering work as seen in Northern Europe as well as recent research published in East, South and Southeast Asia should encourage us to look beyond scholarship written in English. A review of academic articles published in local languages as well as possibly collecting extra empirical data in non-English settings would be critical to fully assessing the reach of automated journalism. Although my corpus is also inclusive of two articles in Spanish that were adapted into English ( Rojas Torrijos, 2019; Túñez-Lopez *et al.*, 2019), an English version of other articles written in this language is unavailable on the publisher's website ( Sánchez Gonzáles & Sánchez González, 2017; Túñez-López *et al.*, 2018). The same goes for articles written in French ( Dierickx, 2018; Dierickx, 2019) and Portuguese ( Carneiro dos Santos, 2016), all of which would be especially worth examining as they open a research window not only in Europe, but also in the Americas and in Africa.

### Reach and practice

**Figure 2.**
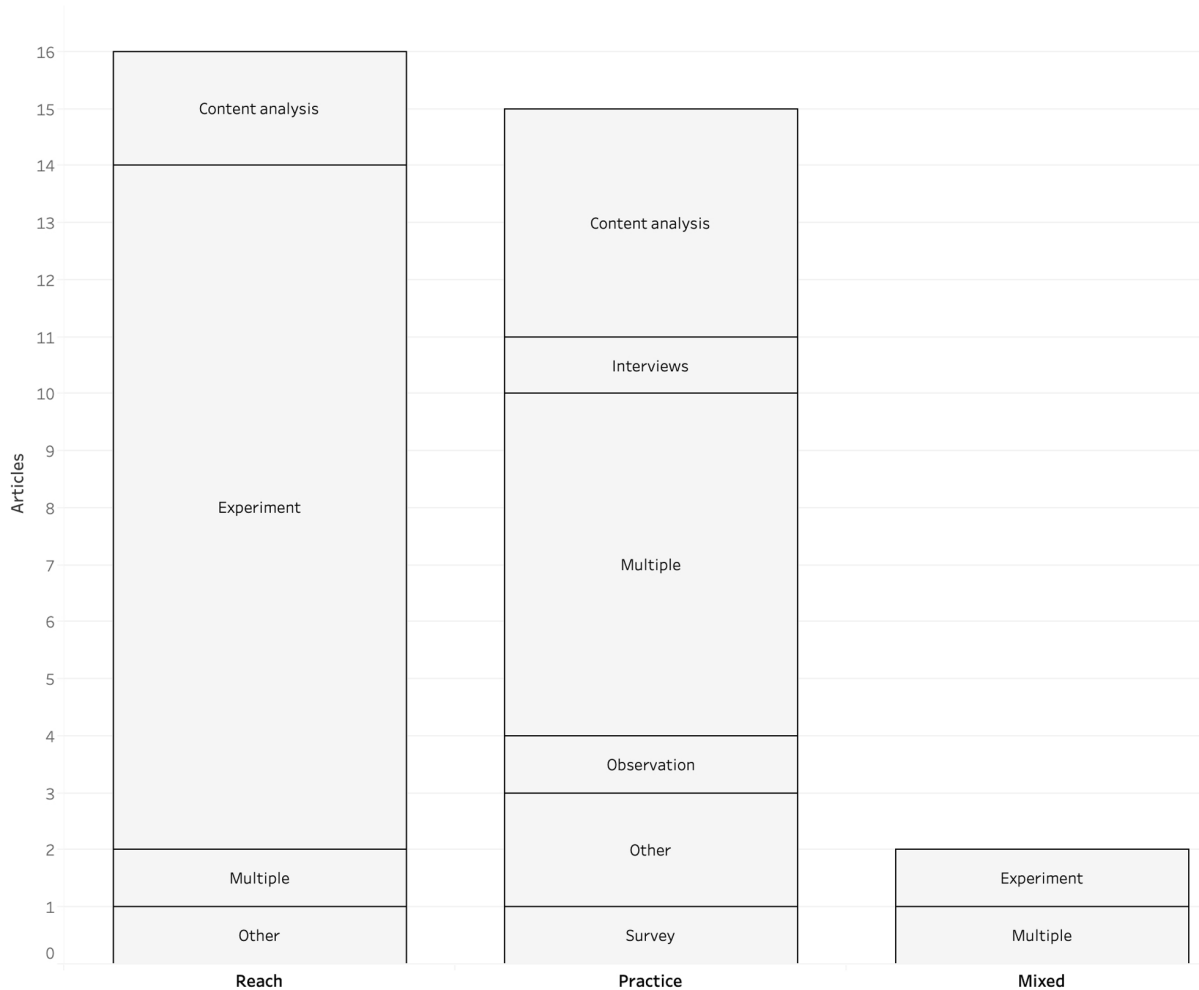
Methods employed for each field of inquiry in corpus.

In a third step, I looked at the fields of inquiry and at the methods used in the scholarship under study, in order to delineate the research orientations that characterise them ( Figure 2). For this purpose, I discerned two main fields of inquiry: first, the reach of automated journalism, which includes studies on the perceptions of news readers (i.e., whether they rank it similarly to human-written content) and those on the wider repercussions of automated journalism, such as its impacts on the legal and financial spheres; second, the practice of automated journalism, which encompasses technically oriented studies looking into its functioning, studies focused on its deployment within news organisations and those focused on implications for media labour and other associated actors ^1^.

Regarding the reach of automated journalism, I found that the articles focusing on the perceptions of the technology were almost entirely constituted of experiments conducted on readers. These experiments are either used solely ( Clerwall, 2014; Graefe *et al*., 2018; Haim & Graefe, 2017; Liu & Wei, 2019; Melin *et al*., 2018; Tandoc *et al.*, 2020; Waddell, 2018; Waddell, 2019a; Waddell, 2019b; Wölker & Powell, 2018; Wu, 2020; Zheng *et al.*, 2018) or in combination with other methods ( Kim & Lee, 2018). In line with Graefe’s & Bohlken’s findings (2020), they highlight in great part that readers evaluate the objectivity, trustworthiness and credibility of automated journalism as similar to those of human journalists, although when reading for pleasure, readers tend to prefer human-written content ( Graefe *et al*., 2018; Haim & Graefe, 2017; Melin *et al*., 2018; Zheng *et al.*, 2018). Besides, a growing stream of research is looking at combined authorship (humans and algorithms), which has demonstrated promising results so far regarding this method of co-creation ( Tandoc *et al.*, 2020; Waddell, 2019a; Waddell, 2019b; Wölker & Powell, 2018). 

Besides analysing readers’ perceptions, articles focusing on the reach of automated journalism are also concerned with the larger repercussions of the technology. Lewis *et al.* (2019) as well as Díaz-Noci (2020) both resorted to content analysis to analyse the legal impacts of automated journalism. Lewis, Sanders and Carmody argue that media organisations could potentially be condemned for negligence when “defamatory content slips through the cracks” (p. 15). For his part, Díaz-Noci notes that human intervention in the creation of automated news could help news organisations secure copyright over this type of content. In the business sphere, Blankespoor *et al.* (2018) conducted a series of quantitative tests to analyse the market effects of automated journalism, which showed a correlation between the automation of financial news and an increase in trading volumes for firms less covered prior to automation. However, no impact on determining trade values was found.

In contrast to scholarship concerned with the reach of automated journalism, those focusing on its practice involve many methods, most of the time combined together. I found that technically oriented studies look at the functioning of the technology to demonstrate its potential as well as its limitations. Caswell & Dörr (2018) carried out a series of tests with an NLG software in an attempt to create automated news that would be more complex than existing articles. They hold that reporting on events in a database format (i.e., “structured journalism”) makes it possible for uncomplicated stories such as car chases to be automated, but not as far as complicated stories such as parliamentary proceedings are concerned. In another technically oriented article, Visvam Devadoss *et al.*, (2019) managed to create a fully operational NLG system able to draw on materials collected online and on social media to feed a news website.

Another area of investigation pertaining to the practice of automated journalism has to do with organisational impacts on newsrooms. In the realm of sports journalism, two separate content analyses found, on the one hand, that commonalities between automated and human-written output prevailed over differences ( Túñez-Lopez *et al.*, 2019) and, on the other hand, that human intervention in the editorial process remained important ( Rojas Torrijos, 2019). Regarding the use of “news bots” and “chatbots,” ^2^ a digital ethnography established that news bots could help media outlets reach out to a niche and geo-specific audience, but sometimes lack data transparency ( Lokot & Diakopoulos (2016). Similarly, a content analysis combined with interviews revealed that chatbots can be used to appeal to new audiences, but that they also need to be scrutinised and to be made accountable in order to maintain public media values ( Jones & Jones, 2019). In addition, another content analysis combined with interviews showed discrepancies in attribution bylines, which led the authors to suggest a comprehensive attributing policy that covers, on the one hand, stories generated through algorithms only, and on the other hand, news articles co-developed by humans and algorithms ( Montal & Reich, 2017).

A third and last domain that relates to automated journalism practices is research that looks at individual impacts on media labour and other associated actors. These studies include first-hand accounts gathered through interviews ( Lindén, 2017), surveys ( Kim & Kim, 2017) or by combining multiple methods that all include interviews ( Dörr, 2016; Kim & Kim, 2018; Thurman *et al.*, 2017; Young & Hermida, 2015). They either highlight a change in newsroom dynamics with established actors such as crime journalists progressively losing their influence to a “new class of computational journalist and non-human journalist” ( Young & Hermida, 2015, p. 393), the assumption that journalists will be able to mitigate disruptional changes brought on by automation by showing commitment to their jobs ( Lindén, 2017), the conviction among media practitioners that automated journalism is undermined by “fundamental limitations” ( Thurman *et al.*, 2017) or the psychological traits journalists adopt when faced with the technology ( Kim & Kim, 2018). This stream of research also examines the dominating mindset among media executives, who would reportedly lean toward the implementation of automated journalism instead of hiring additional journalists ( Kim & Kim, 2017), and delineates the market position of automated journalism through interviews with NLG providers ( Dörr, 2016).

In parallel to these first-hand accounts, two studies engage with qualitative content analysis to examine the collective impacts of automated journalism on media practitioners and other associated actors. In the earliest study on automated journalism found, van Dalen (2012) suggests – after having scrutinised 68 blog posts and newspaper articles – that journalists could react to the introduction of the technology by emphasizing their very human skills (e.g., creativity, personality). He also stresses that routine tasks could be assigned to automated news, so that humans could focus on more demanding formats (provided that newsrooms do not fail to reassign them). Likewise – after having examined 63 pieces of media content, websites and blogs – Carlson (2015) concludes that automated journalism could as much be used to alleviate or augment the work of media practitioners as it could be used to make them redundant. However, these studies investigating broader impacts on media labour and other associated actors were published at an early stage of newsroom adoption of automated journalism, and as such can be considered as being exploratory.

In conclusion, research on the reach of automated journalism showed that readers perceive it similarly to human output for credibility, objectivity and trustworthiness, but not as far as reading for pleasure is concerned. Future research should therefore evaluate the latest breakthroughs in NLG production against this criterion. As for the larger repercussions of automated journalism, it would be worth continuing to look at the influence the technology has on other domains, such as on the legal industry ( Monti, 2019; Weeks, 2014). For research dealing with practice, scholarship on the functioning of automated journalism, as well as those focused on organisational impacts, revealed that the technology provides great affordances but should also be weighed against transparency and accountability requirements. That being said, I was unable to draw any definite conclusion with regard to research on individual impacts on media practitioners and other associated actors, while research on the collective impacts of the technology can be considered to be too exploratory. This calls for further research into this area, so as to detect common patterns across media organisations.

### Underexploited sociological frameworks

**Figure 3.**
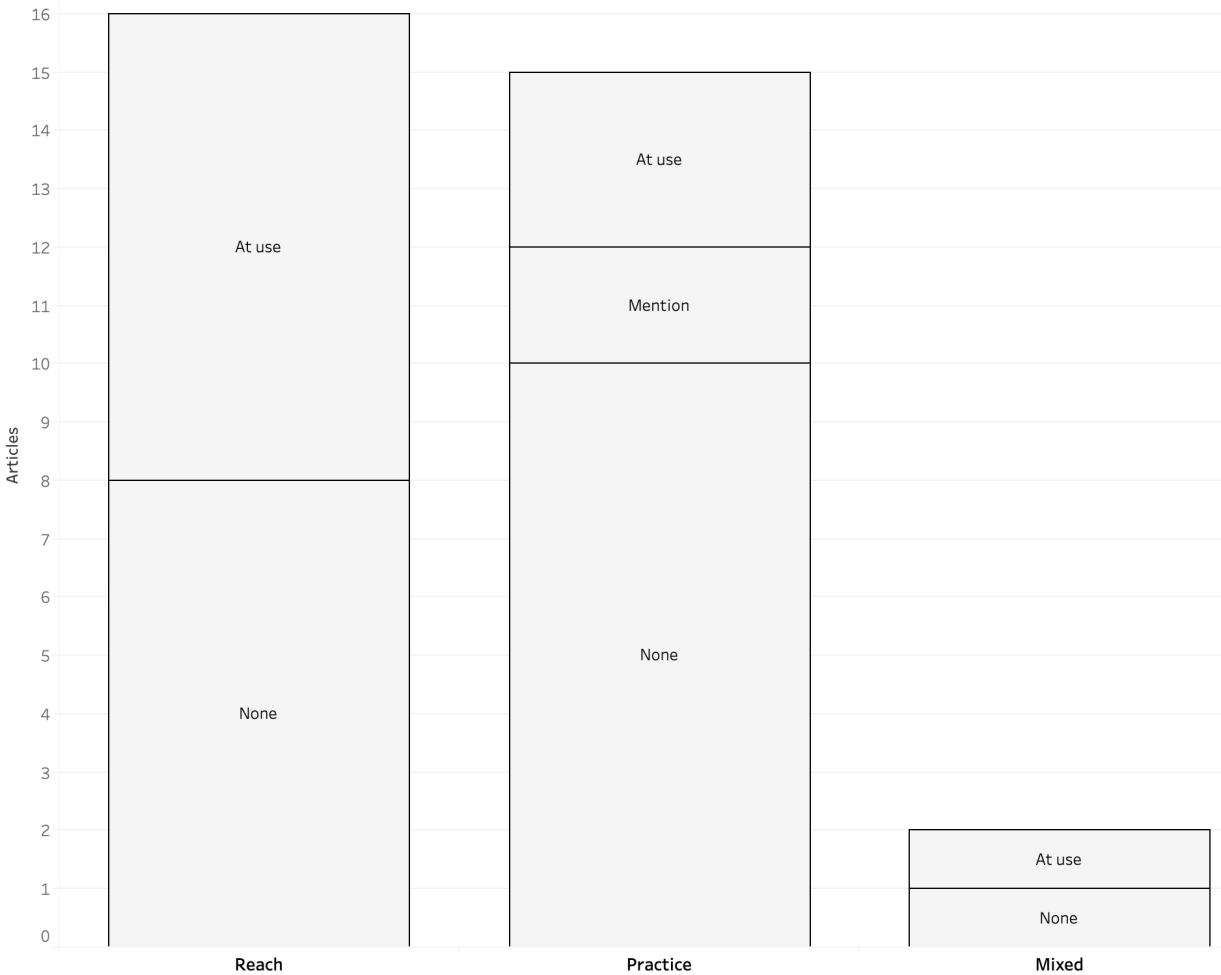
Use of theory for each field of inquiry in corpus.

Finally, I examined my corpus to see if theory was used, and then compared these results with the fields of inquiry ( Figure 3). In most cases, no theory was employed, which is especially valid for studies that focused on the practice of automated journalism, with only three articles actively making use of a theoretical framework ( Dörr, 2016; Kim & Kim, 2017; Kim & Kim, 2018) and two building on theory, but not operationalising it in the variables they used ( Carlson, 2015; Lindén, 2017). By contrast, two thirds of the articles that made an active use of theory focused on the reach of automated journalism and dealt with evaluating readers’ perceptions ( Haim & Graefe, 2017; Liu & Wei, 2019; Tandoc *et al.*, 2020; Waddell, 2018; Waddell, 2019a; Waddell, 2019b; Wu, 2020; Zheng *et al.*, 2018).

**Figure 4.**
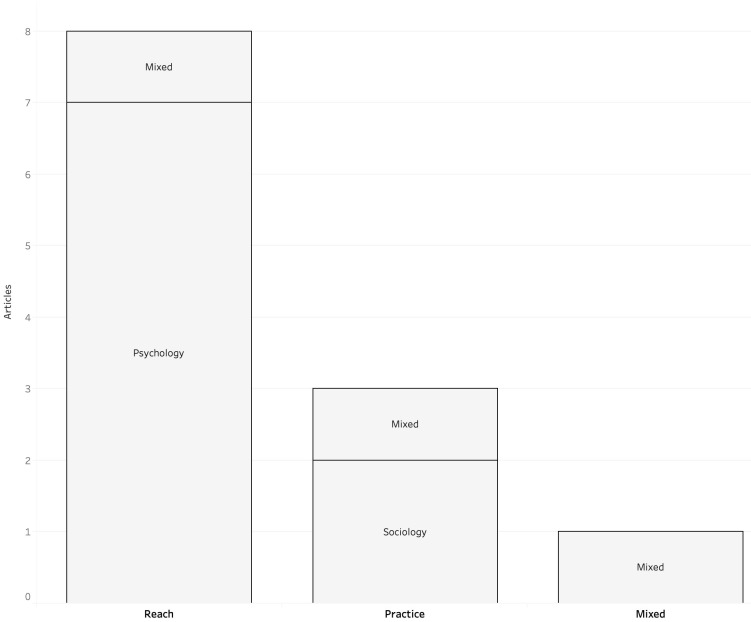
Theoretical backgrounds for each field of inquiry in corpus.

When comparing the theoretical backgrounds of articles actively using theory with the fields of inquiry ( Figure 4), I found that those concerned with readers’ perceptions resorted to theories belonging to the realm of psychology (i.e., expectancy violations theory in Liu & Wei, 2019; Waddell, 2018; Waddell, 2019a; Tandoc *et al.*, 2020; MAIN model in Waddell, 2018; Waddell, 2019b; expectation-confirmation theory in Haim & Graefe, 2017; cognitive authority theory in Wu, 2020; similarity attraction in Waddell, 2019b), except for one article using a mixed sociological-psychological framework (i.e., high-context/low-context cultures and holistic/analytic thinking framework in Zheng *et al.*, 2018), while two of the three studies that actively employed theory to analyse the practice of automated journalism used sociological frameworks to investigate it with a focus on media labour (i.e., institutionalism in Dörr, 2016; institutional entrepreneurship, structural inertia, and institutional isomorphism in Kim & Kim, 2017), with the third one using a mixed sociological-psychological framework for the same purpose (i.e., innovation resistance theory and institutionalism in Kim & Kim, 2018) ^3^.

In order to have a better idea of the sources that inspired these theoretical considerations, I then looked at the most-cited references throughout the entire corpus ( Table 2). First, I noticed that the most-cited sources had to do with empirical studies published on automated journalism, whether they were the same journal articles that I systematically retrieved ( Caswell & Dörr, 2018; Carlson, 2015; Clerwall, 2014; Dörr, 2016; Graefe *et al*., 2018; Jung *et al.*, 2017; Lindén, 2017; Lokot & Diakopoulos, 2016; Montal & Reich, 2017; Thurman *et al.*, 2017; van Dalen, 2012; Waddell, 2018; Young & Hermida, 2015) or other forms of publication that I did not select such as a report ( Graefe, 2016), a conference paper ( van der Kaa & Krahmer, 2014) and a book chapter ( Lemelshtrich Latar, 2015). In addition to this, other empirical materials that did not directly deal with automated journalism, but rather with algorithmic accountability ( Diakopoulos, 2015), the use of artificial intelligence for investigative reporting ( Broussard, 2015), computational journalism ( Karlsen & Stavelin, 2014; Stavelin, 2013) and archival research looking into the evolution of technologically specific forms of work ( Powers, 2012) also figured in this listing.

**Table 2.** Most-cited references † in corpus.

Number of citations	Publications
**24**	Clerwall (2014)
**21**	Carlson (2015)
**20**	van Dalen (2012)
**17**	Anderson (2012)
**16**	Graefe (2016)
**15**	Dörr (2016); Young & Hermida (2015)
**14**	Graefe *et al.* (2018); van der Kaa & Krahmer (2014)
**12**	Flew *et al.* (2012)
**11**	Coddington (2015); Diakopoulos (2015); Lemelshtrich Latar (2015)
**10**	Thurman *et al.* (2017)
**9**	Gillespie (2014); Napoli (2014)
**8**	Lokot & Diakopoulos (2016); Sundar & Nass (2001)
**7**	Dörr & Hollnbuchner (2017); Hamilton & Turner (2009); Montal & Reich (2017)
**6**	Caswell & Dörr (2018); Gynnild (2014); Levy (2012); Lewis & Westlund (2015a); Lindén (2017); Meyer (1988); Sundar (1999); Sundar (2008)
**5**	Appelman & Sundar (2016); Broussard (2015); Cohen *et al.* (2011); Deuze (2005); Hovland & Walter (1951); Jung *et al.* (2017); Karlsen & Stavelin (2014); Lewis & Westlund (2015b); Pavlik (2000); Powers (2012); Reiter & Dale (2000); Stavelin (2013); Waddell (2018)

† Only citations mentioned 5 times or more are indicated.

Theoretical contributions published in an academic publication ^4^ were in great part concerned with exploring sociological aspects that relate to news making as well as to digital and algorithmic transformations ( Anderson, 2012; Coddington, 2015; Deuze, 2005; Dörr & Hollnbuchner, 2017; Flew *et al.*, 2012; Gillespie, 2014; Gynnild, 2014; Lewis & Westlund, 2015a; Lewis & Westlund, 2015b; Napoli, 2014; Pavlik, 2000). That being said, the suggested sociological lenses some of these publications put forward remained largely unexploited in the articles I analysed; even if a handful of studies on the practice of automated journalism put these contributions at the centre of their analysis: (i.e., Anderson, 2012 and Napoli, 2014 in Dörr, 2016; Anderson, 2012 in Young & Hermida, 2015; Lewis & Westlund, 2015a in Thurman *et al.*, 2017; Deuze, 2005 in Lindén, 2017), none of them operationalised them as variables.

Out of all the theoretical contributions that were dealing with sociological aspects, only a handful advised to engage with well-established theoretical frameworks to look at how algorithms are transforming journalism. Dörr & Hollnbuchner (2017) recommended using traditional theories of ethics (i.e., deontology, utilitarianism, virtue ethics and contractualism) while Napoli (2014) pointed at institutional theory to emphasise how a social constructivist approach as well as a focus on the concept of Institutional isomorphism could help investigate algorithmic media consumption and production. Along with the same institutional lenses, Anderson (2012) recommended using Bourdieu’s field theory to add “a vector of power dynamics” to the field of technological innovation, which he described (p. 1013) as being “too often understood from within an “all boats will rise” mentality.” Although institutionalism is actively used – either partly or entirely – in a few studies on professionals ( Dörr, 2016; Kim & Kim, 2017; Kim & Kim, 2018), the Bourdieusian lenses suggested by Anderson remained unexplored in the publications I systematically retrieved. Outside the corpus, though, Field theory is at the heart of Wu’s *et al.* (2019a) of how algorithmic automation reshapes the journalistic field. 

Finally, a last strand of studies visible through my listing of most-cited references are contributions focusing on readers’ perceptions and evaluation of credibility, whether they focus on communication materials overall ( Hovland & Weiss, 1951), newspapers ( Meyer, 1988), printed and online news ( Sundar, 1999), online news only ( Sundar & Nass, 2001), technological aspects of digital media ( Sundar, 2008) or message content ( Appelman & Sundar, 2016). Contrarily to the sociological frameworks that I mentioned above, these psychologically-inspired studies – which can be empirical or theoretical – are largely operationalised as variables in the articles that investigated readers’ perceptions ( Sundar, 1999 in Clerwall, 2014, Haim & Graefe, 2017, Kim & Lee, 2019, Graefe *et al.*, 2018, Melin *et al.*, 2018 and Wu, 2020; Sundar, 2008 in Waddell, 2018, Waddell, 2019b and Wu, 2020; Sundar & Nass, 2001 in Zheng *et al.*, 2018; Meyer, 1988 in Liu & Wei, 2019, Wölker & Powell, 2018, Tandoc *et al.*, 2020 and Wu, 2020; Appelman & Sundar, 2016 in Waddell, 2018, Waddell, 2019a, Waddell, 2019b and Liu & Wei, 2019).

In sum, although studies that adopt psychological approaches to focus on readers' perceptions of automated journalism tend to fully engage with theory, the active use of sociological frameworks to investigate media labour remains largely unexploited. Of the multitude of theoretical contributions dealing with sociological aspects that have been mentioned, only a couple engage with well-established theories that can be employed to investigate automated journalism practices: Institutional theory, which is actively used in three studies ( Dörr, 2016; Kim & Kim, 2017; Kim & Kim, 2018), and Field theory, which, so far, remains unused to study automated journalism only. 

## Conclusions

In this article, I analysed some of the features of a selection of scholarship on automated journalism in order to contribute a research agenda that advances guidelines and suggestions for future investigations into this area. First of all, I noticed that, although the expression “robot journalism” has largely been called into question, there is little debate as to whether “automated journalism” should be preferred, even if it could also refer to a wide range of automated tasks within newsrooms. Second, I became conscious of the fact that limiting myself to scholarship written in English prevents me from engaging with the full picture of automated journalism, and called for a review of scholarship published in other languages, such as in French, Spanish and Portuguese as they open a research window in Europe, the Americas and Africa, but also in local Asian languages, especially in order to better understand the strategies developed in a country like China. Third, I demonstrated the need to take a better look at individual and collective impacts the technology has on media practitioners and other associated actors to discern common patterns across media organisations, while studies on readers predominantly showed that they perceive human-written content and automated content in similar ways. Lastly, I found that, although psychologically-inspired frameworks were well exploited in studies focusing on readers’ perceptions, sociological lenses were, in comparison, underused to look at automated journalism practices.

On a last note, while looking at the use of well-established theories to investigate automated journalism with a focus on media labour, I observed that Institutionalism was actively used in a couple of studies, but that the Bourdieusian lenses that Anderson suggested (2012) were missing. Therefore, the framework provided by field theory could be worth exploring in future studies exploring the individual and collective impacts the technology has on media labour, to find common patterns across media organisations.

## Data Availability

Zenodo: Supporting dataset – A systematic review of automated journalism scholarship: guidelines and suggestions for future research https://doi.org/10.5281/zenodo.4442328 ( Danzon-Chambaud, 2021a) This project contains the following underlying data: Dataset_Systematic_Review_Automated_Journalism.xlsx (This dataset supports the following article, conditionally accepted for publication in Open Research Europe: "A systematic review of automated journalism scholarship: guidelines and suggestions for future research.") Data are available under the terms of the Creative Commons Attribution 4.0 International license (CC-BY 4.0). Zenodo: PRISMA checklist for ‘A systematic review of automated journalism scholarship: guidelines and suggestions for future research’. https://doi.org/10.5281/zenodo.4444842 ( Danzon-Chambaud, 2021b) Zenodo: PRISMA flow diagram for 'A systematic review of automated journalism scholarship: guidelines and suggestions for future research'. https://doi.org/10.5281/zenodo.4444916 ( Danzon-Chambaud, 2021c) Data are available under the terms of the Creative Commons Attribution 4.0 International license (CC-BY 4.0).
