# Supplementary material for: A systematic review of automated journalism scholarship: guidelines and suggestions for future research
Source: Open Res Eur. 2021 Mar 24;1:4. [Version 1] doi: 10.12688/openreseurope.13096.1 (PMC10445913; doi:10.12688/openreseurope.13096.1)
Supplement: Peer review history

# Peer review: Reviewer response for version 1

**DOI:** 10.21956/openreseurope.14166.r27043

**Published:** 2021-07-23

**Authors:** Jessica Kunert

**Affiliations:** 1 Institute for Journalism and Communication Studies, University of Hamburg, Hamburg, Germany

I would like to thank Samuel Danzon-Chambaud for this thorough review of automated journalism scholarship. He evaluates semantical, chronological and geographical features of the articles and discusses their implications for the future.

 I have a few comments. First, to make the argument for a review of this scholarship even stronger, I suggest adding a few more recent examples of the application in the introduction. Germany, for example, is now using it for election coverage (MDR; federal state elections 2021 in Sachsen-Anhalt) and for sports reporting beyond amateur football (BR; basketball). Not that I personally need that hint that this field of research is crucial (please read this with a wink), but it would give people who are not in the field a better notion of what is going on and why studying automated journalism is important.

 My second, more major suggestion is to add more structure to the article to make it more accessible. While I do think the article is a good read, I’d prefer to see it having information that can be digested (and then cited) at first glance. I feel that in the introduction, when talking about automated journalism being a part of computational studies, there could be more clarity on where the individual research foci are situated. Moreover, one research field is missing, namely the effect on journalistic roles in conjunction with the affordances of automated journalism. Let me shamelessly bring my own work forward here – the Thurman, Dörr, & Kunert 2017 article deals with this, and more recently my 2020 article on automated sports journalism.

 Adding more structure could also mean adding more tables instead of really long sentences. This for example could apply to putting the research aims of ‘practice’ into tables for a better overview (p. 6 to 8). The same goes for the multitude of theories used (p. 9).

 As for the methods, I agree that wading through the scholarship is hard, especially because so many articles do not concern journalism but are rather tech-oriented. Samuel Danzon-Chambaud also says himself later on that focusing only on articles published in English might be a problem – but I don’t see this as a weakness of this article, but rather as a call to action to band together and get an overview of scholarship around the world in international teams. However, I would have liked to see the codebook in full; maybe it’s possible to upload it.

 The results are quite exciting, especially the discussion of the terms. My suggestion would be to add a paragraph on the ‘historical’ use of the terms – ‘robot journalism’ was an early term (quite possibly because there simply wasn’t a better one, at least not such a colourful one), and things are changing rapidly. The latest one I’ve heard is ‘augmented journalism’, referring to the human-machine partnership. Maybe this could be added to give a look into the future? Moreover, I think a key finding is that sociological lenses are under exploited – researchers should definitely read up on theory in this field (but of course, I can also speak for myself).

 One more thing on presentation: It would help immensely if the figures were labelled more clearly, meaning that for example in figure 2, it’s hard to see at first glance just how many studies used content analysis and so on. Adding the n to the individual categories would help the matter.

 In sum, this a valuable article that demonstrates where research has made headway – and where more work needs to be done.

Are sufficient details of the methods and analysis provided to allow replication by others?

Partly

Are the rationale for, and objectives of, the Systematic Review clearly stated?

Yes

Is the statistical analysis and its interpretation appropriate?

Yes

Are the conclusions drawn adequately supported by the results presented in the review?

Yes

Reviewer Expertise:

Automated journalism; computational journalism

I confirm that I have read this submission and believe that I have an appropriate level of expertise to confirm that it is of an acceptable scientific standard.

## References

[1] : Automation in Sports Reporting: Strategies of Data Providers, Software Providers, and Media Outlets. *Media and Communication* .2020;8(3) : 10.17645/mac.v8i3.2996 5-15 10.17645/mac.v8i3.2996

# Peer review: Reviewer response for version 1

**DOI:** 10.21956/openreseurope.14166.r26541

**Published:** 2021-04-27

**Authors:** Colin Porlezza

**Affiliations:** 1 Institute of Media and Journalism, Università della Svizzera italiana, Lugano, Switzerland

In his article, Danzon-Chambaud offers a thorough overview of the latest empirical research on automated journalism published in peer-reviewed journals. The rationale for looking into this particular topic relates to two aspects: first, AI and automated journalism have become quite common in (elite) news organizations around the world. Second, as a consequence, this phenomenon has come under close scrutiny of journalism studies as well.

 The contribution carried out a systematic literature review by selecting articles based on empirical research on automated journalism. The articles had to be specifically about automated journalism and not generally about AI and journalism, which means that only articles dealing either with “the process of using software or algorithms to automatically generate news stories without human intervention – after the initial programming of the algorithm” (Graefe, 2016, p. 14) ^1^, or the auto generation of journalistic texts were taken into account. In a second step, the author carried out a descriptive analysis of the specific terms used when referring to automated journalism, the chronological and geographical traits, the fields of inquiry and the methods used in the scholarship, and whether and which theories the articles were using.

 The article offers a useful and thorough insight into a quickly growing field of enquiry and is therefore a good starting point if scholars want to familiarize themselves - not necessarily with the findings of the empirical studies - but with the way digital journalism studies tried to tackle the issue of automated journalism. It analyzes therefore the characteristics of research into automated journalism to identify dominant patterns and themes regarding semantics, methods and theories. This article is a first of its kind, which makes for a relevant contribution because - as the article demonstrates - the theoretical approaches used in the investigations are limited.

 I would like to focus on two specific issues: first, the semantic question whether the expression automated journalism is too narrow and does not reflect media practitioners’ perspectives. And second, a discussion about what kind of theories to use in the future, and why.

 Let’s start with the first question: the author is certainly right in stating that automated journalism has been used in a rather narrowish way. By looking at the publications in the field it becomes quite clear that automated journalism is the preferred “term within industry and academia” (Danzon-Chambaud 2021). However, there has not been a coherent use and application of the different terms in the field, and Wu’s *et al*. (2019, p. 1453) ^2^ observation that the expression “does not reflect media practitioners' views” is equally true. The main reason for this incoherence - we could also characterize it in a more positive way as a plurality - shows first of all a high vitality in the field. As Feola (2015) ^3^ states, this is quite common in the social sciences, where different paradigms and concepts coexist at a time. Particularly in a new and emerging field of research such as this one, it might also be argued that a plurality of expressions “with ‘loose’ meanings that also benefit from a strong metaphoric power can be highly effective to stimulate research and action, and to create a much needed common ground for scholars from different disciplines, as well as among scientists and stakeholders, which facilitates inter- and transdisciplinarity” (Feola, 2015). A complex field such as the one revolving around automated journalism is characterized by increasing interdisciplinarity, and therefore needs not only to assess the conceptual diversity, but also the different concepts’ blind spots as well as the different research approaches. The challenge therefore is to find a useful and creative way “to foster a dialog around the potential and complementarities of different concepts: what concepts can be applied to study different types of systems, what prescriptive assumptions inform them, what concepts connect with what research paradigm (…)” (idem). What is needed - and in this I convene with the Danzon-Chambaud’s critique - is a more structured engagement with the different concepts, but also a critical discussion that includes different disciplines and traditions of thought. A good example of such a reflective contribution can be seen in Lewis, Guzman and Schmidt’s (2019) ^4^ paper on “Automation, Journalism, and Human–Machine Communication”, an article suggesting that “research focused on automated journalism, has much to learn from Human-Machine Communication (HMC)” - which is a theoretical paper and therefore not included in the author’s review. Hence, for a more complete overview of the field’s engagement with the phenomenon of automated journalism, it might have proven helpful to include theoretical contributions in the review as well.

 This brings me to the second point of my review, that is, the question of what kind of theories to use in the future. At the very end of his article, the author states “while looking at the use of well-established theories to investigate automated journalism with a focus on media labour, I observed that Institutionalism was actively used in a couple of studies, but that the Bourdieusian lenses that Anderson suggested (2012) were missing. Therefore, the framework provided by field theory could be worth exploring in future studies exploring the individual and collective impacts the technology has on media labour, to find common patterns across media organisations” (Danyon-Chambaud 2021). While the author is certainly right in stating that a field theory perspective has not been used frequently, it is nevertheless surprising that - given the increasingly interdisciplinary character of the field - there are no references to other promising theoretical frameworks such as Human-Machine-Communication (HMC), Actor-Network-Theory (ANT) or Science and Technology Studies (STS) precisely when it comes to future endeavors in the field. Especially when it comes to questions of power shifts between humans and technology, field theory is not the only framework able to “add an additional vector”, but HMC for instance is equally suited to investigate the social positioning of technology by looking at “how a person interprets what a particular technology is in relation to themselves, the factors contributing to such interpretations, and, in turn, how such conceptualizations inform their interactions” (Guzman & Lewis, 2019) ^5^. If the author chooses to point out a Bordieuan lens, it would have been useful to offer more room to the author’s explanations given the many theoretical alternatives.

 All in all, the article is a helpful snapshot of how the field of digital journalism studies engages with the emergent phenomenon of automated journalism. The findings could also be used to initiate a wider discussion on the current plurality of concepts and on how to deal with the differences, complementarities and blind spots of the theoretical frameworks, particularly as the field becomes more interdisciplinary.

Are sufficient details of the methods and analysis provided to allow replication by others?

Yes

Are the rationale for, and objectives of, the Systematic Review clearly stated?

Yes

Is the statistical analysis and its interpretation appropriate?

Yes

Are the conclusions drawn adequately supported by the results presented in the review?

Partly

Reviewer Expertise:

Digital journalism studies; artificial intelligence; media accountability and transparency; journalism innovation.

I confirm that I have read this submission and believe that I have an appropriate level of expertise to confirm that it is of an acceptable scientific standard.

## References

[1] : Guide to Automated Journalism. *Tow Center for Digital Journalism, Columbia University* .2016;

[2] : A Field Analysis of Journalism in the Automation Age: Understanding Journalistic Transformations and Struggles Through Structure and Agency. *Digital Journalism* .2019;7(4) : 10.1080/21670811.2019.1620112 428-446 10.1080/21670811.2019.1620112

[3] : Societal transformation in response to global environmental change: A review of emerging concepts. *Ambio* .2015;44(5) : 10.1007/s13280-014-0582-z 376-90 10.1007/s13280-014-0582-z 25431335PMC4510318

[4] : Automation, Journalism, and Human–Machine Communication: Rethinking Roles and Relationships of Humans and Machines in News. *Digital Journalism* .2019;7(4) : 10.1080/21670811.2019.1577147 409-427 10.1080/21670811.2019.1577147

[5] : Artificial intelligence and communication: A Human–Machine Communication research agenda. *New Media & Society* .2020;22(1) : 10.1177/1461444819858691 70-86 10.1177/1461444819858691
